# The Impact of Preoperative and Postoperative Malnutrition on Outcomes for Ampullary Carcinoma After Pancreaticoduodenectomy

**DOI:** 10.3389/fonc.2021.748341

**Published:** 2021-11-30

**Authors:** Jikuan Jin, Guangbing Xiong, Xiaoxiang Wang, Feng Peng, Feng Zhu, Min Wang, Renyi Qin

**Affiliations:** Department of Biliary-Pancreatic Surgery, Tongji Hospital, Tongji Medical College, Huazhong University of Science and Technology, Wuhan, China

**Keywords:** pancreatoduodenectomy, postoperative outcomes, malnutrition, Patient-Generated Subjective Global Assessment, ampullary carcinoma

## Abstract

**Purpose:**

The aim of this study was to investigate the effect of preoperative and postoperative malnutrition on postoperative short- and long-term outcomes for ampullary carcinoma after pancreatoduodenectomy (PD).

**Methods:**

Data were collected retrospectively from 511 patients with ampullary carcinoma who underwent PD between June 2012 and June 2019. Nutritional status before and at 3, 6, and 12 months after operation was assessed by the scored Patient-Generated Subjective Global Assessment (PG-SGA). The patients were classified into well-nourished, moderately malnourished, and severely malnourished group according to the PG-SGA score. Propensity score matching (PSM) was performed to adjust baseline characteristics between preoperative group A (well-nourished and moderately malnourished group) and group B (severely malnourished group). After PSM, clinicopathological variables and postoperative complications were compared between the two groups. Univariate and multivariate Cox analysis was also conducted to investigate the prognostic factors of overall survival of patients with ampullary carcinoma who underwent PD.

**Results:**

Preoperatively, 122 (23.9%) patients were classified into well-nourished group, 189 (37.0%) into moderately malnourished group, and 200 (39.1%) into severely malnourished group. After PSM analysis, the incidence of overall postoperative complications was higher in group B than that in group A (50.5% vs. 32.5%, p < 0.001). Multivariate Cox proportional hazards regression model showed that severe malnutrition (PG-SGA score >9 scores) before operation [hazard ratio (HR) = 1.508; 95% CI, 1.103–2.061; p = 0.01] and at 6 months (HR = 4.148; 95% CI, 2.523–6.820; p < 0.001) and 12 months (HR = 5.272; 95% CI, 3.630–7.656; p < 0.001) after operation was an independent prognostic factor of patients who underwent PD for ampullary carcinoma.

**Conclusions:**

Severe malnutrition before and at 6 and 12 months after operation significantly affects the long-term survival of patients with ampullary carcinoma who underwent PD. Additionally, the preoperative malnutrition was closely related to postoperative complications.

## Introduction

Ampullary carcinoma is a relatively rare malignant tumor, associated with a more favorable prognosis than adenocarcinomas of the adjacent pancreatic duct, common bile duct, or duodenum (1). Pancreatoduodenectomy (PD) was widely considered a safe, potentially curative surgical treatment method for ampullary neoplasm (2–4). As reported previously, many factors affect the postoperative outcomes of ampullary carcinoma after PD, including surgical technique factors (5, 6) and host- and disease-related risk factors (7, 8), such as advanced age, comorbidities, jaundice, advanced tumor stage, and adjuvant chemotherapy (9, 10).

Malnutrition was a common problem in patients with malignant tumor and closely related to the immunosuppression and inflammatory activity (11–14), also considered as an important risk factor of postoperative outcomes (7, 13, 15). Numerous studies have revealed that preoperative malnutrition increased the incidence of postoperative complications and affected the long-term survival of patients (13, 15, 16). However, the studies examining whether postoperative malnutrition affects the long-term survival of patients who underwent PD for ampullary carcinoma were lacking. The scored Patient-Generated Subjective Global Assessment (PG-SGA) proposed by Ottery (17), as a noninvasive nutrition measurement tool for oncology patients, was a modification of the Subjective Global Assessment (SGA) (18). The preoperative scored PG-SGA has been validated in colectomy and hepatectomy in many countries (19–21); however, the correlation of postoperative malnutrition assessed by the scored PG-SGA and postoperative outcomes in patients of ampullary carcinoma following PD is still unknown.

The aim of this study was to investigate the effect of preoperative and postoperative malnutrition on postoperative outcomes for ampullary carcinoma after PD.

## Materials and Methods

### Patients

**Figure 1:**
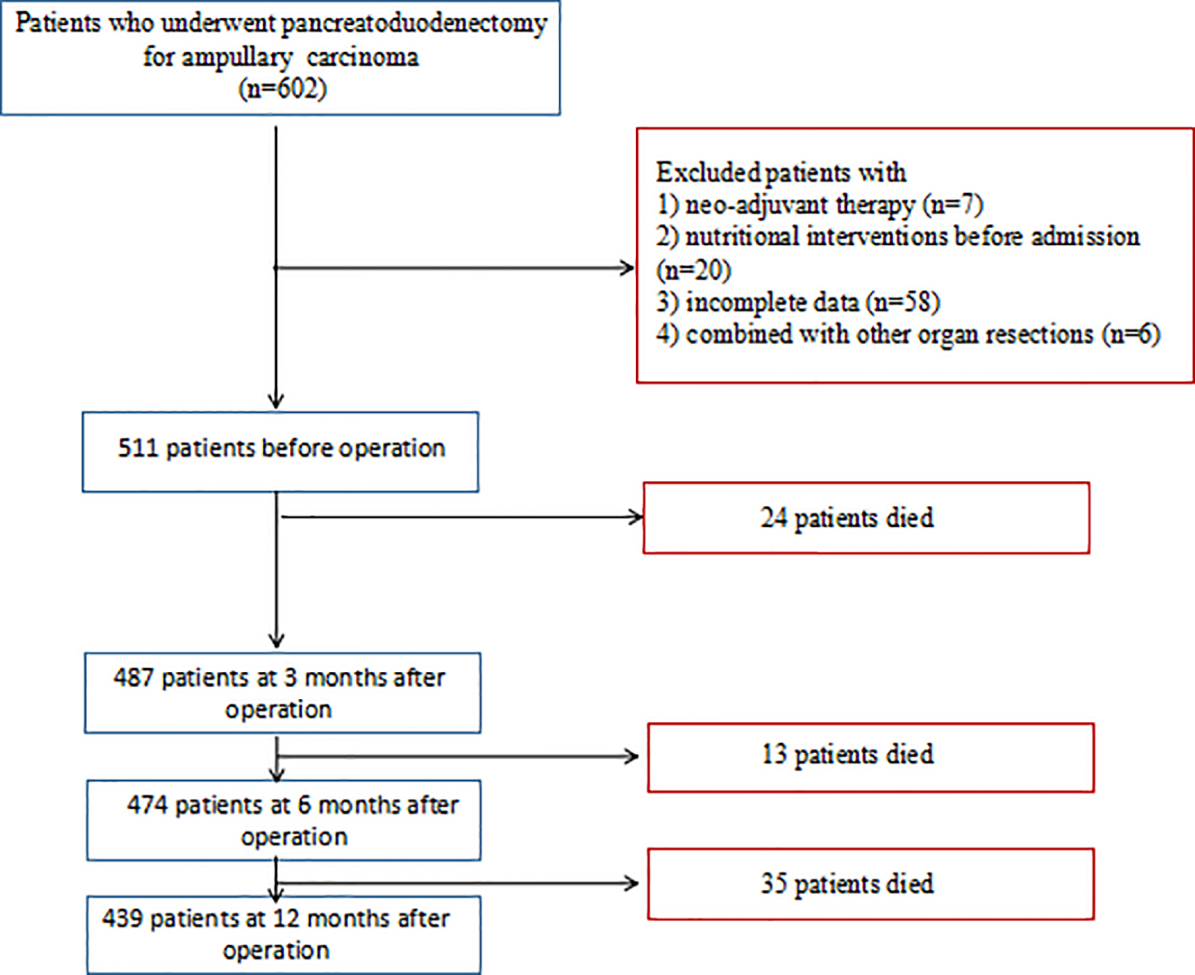
Flow diagram of before surgery and at 3, 6, and 12 months after pancreatoduodenectomy.

Patients who underwent PD for ampullary carcinoma at the Tongji Medical Hospital of Tongji Medical College of Huazhong University of Science and Technology, between June 2012 and June 2019, were retrospectively identified. Individuals with incomplete clinical data, neoadjuvant treatment, nutritional interventions before admission, and combined with other organ resections (such as hepatectomy or colectomy) were excluded from this study (**Figure 1**). After discharge, adjuvant chemotherapy was administered to all patients unless the patient was lost to follow-up or for other reasons. Regimens utilized for ampullary cancer were typically either S1-based or gemcitabine (Gem)-based. Patients were followed up every 3 months for the first 2 years and then every 6 months for a total of 5 years. The enrolled patients were followed until death or June 30, 2021, whichever came first. All patients provided written informed consent to participate in the study, and the study was approved by the Ethics Committee of the local hospital (no. TJ-IRB20190418).

### Nutritional Assessment

The nutritional status of the patients in this study was evaluated by the scored Patient-Generated Subjective Global Assessment (PG-SGA) technique (17), performed by an experienced physician within the initial 24 h of hospitalization and at 3, 6, and 12 months after operation. The scored PG-SGA comprises a patient-generated and a professional component. The scored PG-SGA incorporates a numerical score called the PG-SGA score. The components of the patient generated can be completed by the patient using a check box format, which includes weight change with a score of 0–5 points, food intake with a score of 0–4 points, nutrition effect symptoms with a score of 0–23 points, and activities and functions effected by nutrition with a score of 0–3 points. The professional component was completed by a physician, nurse, or dietitian, which includes disease and age with a score of 0–6 points, the metabolic stress state with a score of 0–9 points, loss of subcutaneous fat with a score of 0–3 points, muscle wasting with a score of 0–3 points, and edema and ascites with a score of 0–3 points. A higher PG-SGA score indicates an increased risk for malnutrition. Patients were divided into three groups according to the PG-SGA score: well-nourished group (with a PG-SGA scores of 0–3 points), moderately malnourished group (with a PG-SGA scores of 4–8 points), and severely malnourished group (with a PG-SGA scores ≥9 points). Then, the well-nourished and moderately malnourished patients were classified into group A; the severely malnourished patients were classified into group B.

### Data Collection

Baseline characteristics were age, sex, body mass index (BMI), comorbidities, American Society of Anesthesiologists (ASA) class, serum albumin, carbohydrate antigen 19-9 (CA19-9), carbohydrate antigen 125 (CA125), and total bilirubin. Pathological diagnoses were T stage, tumor grade, lymph node metastasis, vascular invasion, perineural invasion, surgical margin, perineural invasion, and TNM stage according to the 8th edition of the Cancer Staging Manual of the American Joint Commission on Cancer. The baseline preoperative data was evaluated within 24 h after admission. Intraoperative parameters were operative time and estimated blood loss.

### Definition of Postoperative Outcomes

Major postoperative complications were defined as grade III or higher in the Clavien–Dindo classification (22). For pancreatic fistula, the diagnosis of postoperative pancreatic fistula (POPF) was determined according to the definitions provided by the International Study Group of Pancreatic Surgery (ISGPS) (23). Delayed gastric emptying (DGE) (24), biliary leak (25), and postoperative hemorrhage (PPH) (26) were defined by the definition of the International Study Group of Pancreatic Surgery. Overall survival (OS) was calculated from date of surgery to the date of all-cause death.

### Surgical Procedure

A standard pancreatoduodenectomy was performed by RQ and FZ. For organs resection, the Kocher maneuver was initially performed. The distal stomach was then removed on the left side of the pylorus, and a tunnel was dissected posterior to the pancreatic neck and anterior to the superior mesenteric vein (SMV) and portal vein. Then, the pancreas was transected, the jejunum was transected approximately 15 cm from the ligament of Treitz, cholecystectomy was performed, and the common hepatic duct was transected at the level of the cystic duct. For digestive tract reconstruction, we performed pancreaticojejunostomy by the imbedding pancreaticojejunostomy method, as reported previously (27). Pancreatogastrostomies were performed by embedding the pancreatic remnant into the stomach. An end-to-side hepaticojejunostomy was performed 15 cm away from the pancreaticojejunostomy, an ante colic side-to-side gastroenterostomy was performed with the staple technique, and 2 layers of running 3–0 Vicryl sutures were used to close the gastric stump, 40 cm away from hepaticojejunostomy. Finally, two drains were routinely placed in the area of the pancreatojejunostomy and hepaticojejunostomy, respectively.

### Statistical Methods

Continuous variables were expressed as medians and interquartile ranges (IQRs) and were compared using Mann–Whitney U and Kruskal–Wallis tests. Some variables were dichotomized using median values or normal values. Categorical variables were expressed as frequencies and percentages, and differences in variables between the groups were compared using Pearson’s chi square or Fisher’s exact tests. Survival was estimated with the Kaplan–Meier method and log-rank tests. Cox proportional-hazard regression analysis was used for univariate and multivariate analysis. Variables with *p* < 0.1 on the univariate analysis were included in the multivariate analysis. For all tests, differences with *p* < 0.05 were considered statistically significant. Statistical analysis was performed using the Statistical Package for Social Sciences (SPSS) version 22.0 (IBM Corp., Armonk, NY, USA).

In an attempt to reduce selection bias of baseline characteristics inherent in preoperative group A (well-nourished and moderately malnourished group) and group B (severely malnourished group), the propensity score matched (PSM) analysis [including age, carbohydrate antigen 19-9 (CA19-9), carbohydrate antigen 125 (CA125), T stage, lymph node metastasis, TNM stage, and adjuvant therapy] was performed between the two groups using the one-to-one nearest neighbor method in R Studio version 3.4.3 (R Foundation for Statistical Computing, Vienna, Austria).

## Results

### Patients’ Characteristics

**Figure 2:**
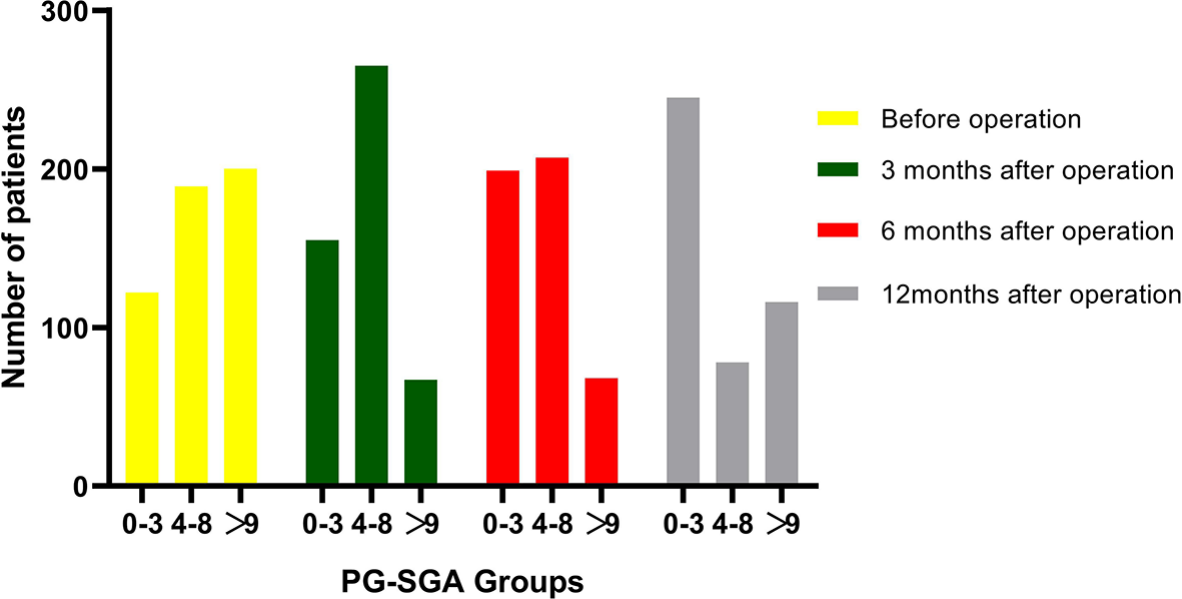
Changes in patients number in PG-SGA groups over time from before to 3, 6, and 12 months after pancreatoduodenectomy. A total of 122, 155, 199, and 245 patients were classified into well-nourished group (0–3 scores) before surgery and at 3, 6, and 12 months after operation, respectively; 189, 265, 207, and 78 patients were classified into moderately malnourished group (4–8 scores) before surgery and at 3, 6, and 12 months after operation, respectively; and 200, 67, 68, 116 patients were classified into severely malnourished group (>9 scores) before surgery and at 3, 6, and 12 months after operation, respectively.

In this study, a total of 511 patients were included in data set before operation. Of the 511 patients, the median age was 58 years (IQR, 49–66 years), namely, 298 (58.3%) male subjects; the median BMI was 21.5 kg/m^2^ (IQR, 19.9–23.5 kg/m^2^). The conclusive stages of the 511 patients who underwent resection according to the AJCC classification were stage I in 195 (38.2%) cases, stage II in 136 (26.6%) cases, and stage III in 180 (35.2%) cases; 218 (42.7%) patients underwent adjuvant chemotherapy. Before operation, 122 (23.9%) patients were classified into well-nourished group, 189 (37.0%) into moderately malnourished group, and 200 (39.1%) into severely malnourished group; the median preoperative PG-SGA score was 8 scores (IQR, 4–11 scores). Furthermore, the patients reduced to 487 patients at 3 months, 474 patients at 6 months, and 439 patients at 12 months after PD (**Figure 1**), and the median PG-SGA scores were 5, 4, and 3 scores at 3, 6, and 12 months after PD, respectively. The clinicopathological characteristics of the patients included in this study are summarized in **Table 1** and **Figure 2**.

**Table 1:** Clinicopathological characteristics of 511 patients who underwent pancreatoduodenectomy for ampullary carcinoma.

Factors	Values
Age, years	58 (49–66)
Sex (male)	298 (58.3)
Body mass index, kg/m2	21.5 (19.9–23.5)
ASA class	
I	75 (14.7)
II	371 (72.6)
III	65 (12.7)
Comorbidities	124 (24.3)
Total bilirubin, μmol/L	73.5 (18.6–163.9)
Albumin, mg/dl	3.7 (3.3–3.9)
CA19-9, U/ml	68 (18–220)
CA125, U/ml	14.1 (9.7–22.5)
Estimated blood loss, ml	200 (100–350)
Operative time, minutes	275 (205–335)
T stage	
T1/T2	268 (52.4)
T3/T4	243 (47.6)
Lymph node metastasis, positive	164 (32.1)
Vascular invasion, positive	37 (7.2)
Perineural invasion	51 (10.0)
Tumor grade	
Well or moderately	414 (81.0)
Poorly	97 (19.0)
Positive resection margin	24 (4.7)
TNM stage	
I	195 (38.2)
II	136 (26.6)
III	180 (35.2)
Adjuvant therapy	218 (42.7)
Preoperative PG-SGA score	8 (4–11)
0–3	122 (23.9)
4–8	189 (37.0)
>9	200 (39.1)
PG-SGA score after operation	
3 months	5 (3–6)
6 months	4 (3–6)
12 months	3 (2–9)

Categorical variables were expressed as n (%); continuous variables are expressed as medians and interquartile ranges (IQR).

ASA, American Society of Anesthesiologists; CA19-9, carbohydrate antigen 19-9; CA125, carbohydrate antigen 125; PG-SGA, Patient-Generated Subjective Global Assessment.

### Comparisons of Clinicopathological Characteristics Between Groups

To control for the effects of confounding factors, propensity score matched analysis was performed to balance the clinicopathological variables between preoperative group A (well-nourished and moderately malnourished group) and group B (severely malnourished group). Before PSM analysis, patients in group B were significantly older (p = 0.021), with a higher total bilirubin (p < 0.001), CA19-9 (p < 0.001), and CA125 level (p = 0.001) and more advanced TNM stage (p = 0.002) and T stage (p = 0.048), and fewer patients received chemotherapy (p = 0.015) than those in group A. After PSM analysis, the baseline characteristics between preoperative groups A and B were adjusted. The results of the analysis of the associations between preoperative nutritional status and clinicopathological characteristics are shown in **Table 2**.

**Table 2:** The relationships between preoperative nutritional status and clinicopathological characteristics in 511 patients underwent pancreatoduodenectomy for ampullary carcinoma after PSM.

Variables	Before PSM			After PSM		
	Well-nourished/moderately malnourished	Severely malnourished	p-value	Well-nourished/moderately malnourished	Severely malnourished	p-value
	N = 311	N = 200		N = 200	N = 200	
Age, years	57 (50–64)	59 (52–65)	0.021	59 (51–64)	59 (52–65)	0.455
Sex (male)	181 (58.2)	117 (58.5)	0.946	118 (59.0)	117 (58.5)	0.919
Body mass index, kg/m2	21.7 (19.9–23.8)	21.8 (19.6–23.4)	0.263	21.7 (19.9–23.7)	21.8 (19.6–23.4)	0.399
ASA class						
I/II	274 (88.1)	172 (86.0)	0.486	173 (86.5)	172 (86.0)	0.885
III	37 (11.9)	28 (14.0)		27 (13.5)	28 (14.0)	
Comorbidities	75 (24.1)	49 (24.5)	0.921	47 (23.5)	49 (24.5)	0.815
Total bilirubin, μmol/L	35.7 (14.0–92.9)	164.7 (82.9–241.0)	<0.001	34.8 (14.5–94.3)	164.7 (82.9–241.0)	0.001
CA19-9, U/ml	48.3 (15.8–149.3)	99.6 (32.4–576.8)	<0.001	78.1 (28.3–249.5)	99.6 (32.4–576.8)	0.114
CA125, U/ml	13.4 (9.1–20.0)	16.0 (10.5–25.3)	0.001	14.4 (9.0–19.0)	16.0 (10.5–25.3)	0.09
T stage						
T1/T2	174 (55.9)	94 (47.0)	0.048	100 (50.0)	94 (47.0)	0.548
T3/T4	137 (44.1)	106 (53.0)		100 (50.0)	106 (53.0)	
Lymph node metastasis, positive	87 (28.0)	77 (38.5)	0.013	71 (35.5)	77 (38.5)	0.534
Vascular invasion	18 (5.8)	19 (9.5)	0.114	12 (6.0)	19 (9.5)	0.191
Perineural invasion	27 (8.7)	24 (12.0)	0.222	25 (12.5)	24 (12.0)	0.879
Tumor grade						
Well/moderately	258 (83.0)	156 (78.0)	0.163	166 (83.0)	156 (78.0)	0.207
Poorly	53 (17.0)	44 (22.0)		34 (17.0)	44 (22.0)	
Positive resection margin	11 (3.5)	13 (6.5)	0.122	9 (4.5)	13 (6.5)	0.380
TNM stage						
I/II	218 (70.1)	113 (56.5)	0.002	124 (62.0)	113 (56.5)	0.263
III	93 (29.9)	87 (43.5)		76 (38.0)	7 (43.5)	
Adjuvant therapy	146 (46.9)	72 (36.0)	0.015	74 (37.0)	72 (36.0)	0.835

Categorical variables were expressed as n (%); continuous variables are expressed as medians and interquartile ranges (IQR).

PSM, propensity score matching; ASA, American Society of Anesthesiologists; CA19-9, carbohydrate antigen 19-9; CA125, carbohydrate antigen 125.

### Postoperative Short-Term Outcomes

In total, 187 patients (36.6%) developed postoperative complications, 98 cases (19.2%) had Clavien–Dindo grade ≥III postoperative complications, namely, pancreatic fistula (n = 92, 18.0%), postoperative bleeding (n = 46, 9.0%), delayed gastric emptying (n = 101, 19.8%), intra-abdominal abscess (n = 46, 9.0%), and bile leakage (n = 9, 1.8%). The median postoperative hospital stay was 21 days (IQR, 16–27 days), and 20 patients (3.9%) died after surgery. After PSM analysis, a higher rate of postoperative complications (50.5% vs. 32.5%, p < 0.001), Clavien–Dindo grade ≥III postoperative complications (28.0% vs. 16.0%, p = 0.004), and delayed gastric emptying (p = 0.005) were more common in group B than in group A. The results of the analysis of the associations between the preoperative nutritional status and postoperative complications are shown in **Table 3**.

**Table 3:** The relationships between preoperative nutritional status and postoperative short outcomes in 511 patients who underwent pancreatoduodenectomy for ampullary carcinoma after PSM.

Variables	Total	Before PSM			After PSM		
		Well-nourished/moderately malnourished	Severely malnourished	p-value	Well-nourished/moderately malnourished	Severely malnourished	p-value
	N = 511	N = 311	N = 200		N = 200	N = 200	
Overall postoperative complications	187 (36.6)	86 (27.7)	101 (50.5)	<0.001	65 (32.5)	101 (50.5)	<0.001
Postoperative complications (CD≥IIIa)	98 (19.2)	42 (13.5)	56 (28.0)	<0.001	32 (16.0)	56 (28.0)	0.004
Pancreatic fistula							
Grade A	61 (11.9)	35 (11.3)	26 (13.0)	0.005	25 (12.5)	26 (13.0)	0.06
Grade B	27 (5.3)	8 (2.6)	19 (9.5)		6 (3.0)	19 (9.5)	
Grade C	4 (0.8)	2 (0.6)	2 (1.0)		2 (1.0)	2 (1.0)	
Bile leakage	9 (1.8)	5 (1.6)	4 (2.0)	0.742	5 (2.5)	4 (2.0)	0.736
Postoperative hemorrhage	46 (9.0)	18 (5.8)	28 (14.0)	0.002	15 (7.5)	28 (14.0)	0.053
Delayed gastric emptying							
Grade A	77 (15.1)	30 (9.6)	47 (23.5)	<0.001	23 (11.5)	47 (23.5)	0.006
Grade B	18 (3.5)	9 (2.9)	9 (4.5)		8 (4.0)	9 (4.5)	
Grade C	6 (1.2)	2 (0.6)	4 (2.0)		1 (0.5)	4 (2.0)	
Intra-abdominal abscess	46 (9.0)	25 (8.0)	21 (10.5)	0.343	18 (9.0)	21 (10.5)	0.613
Mortality	20 (3.9)	7 (2.3)	13 (6.5)	0.016	7 (3.5)	13 (6.5)	0.169
Postoperative hospital stay, days	21 (16–27)	20 (15–27)	22 (18–27)	0.047	20 (15–27)	22 (18–27)	0.149

Categorical variables were expressed as n (%); continuous variables are expressed as medians and interquartile ranges (IQR).

PSM, propensity score matching; CD, Clavien–Dindo classification.

### Overall Survival Analysis According to the Preoperative and Postoperative Nutritional Status

**Figure 3:**
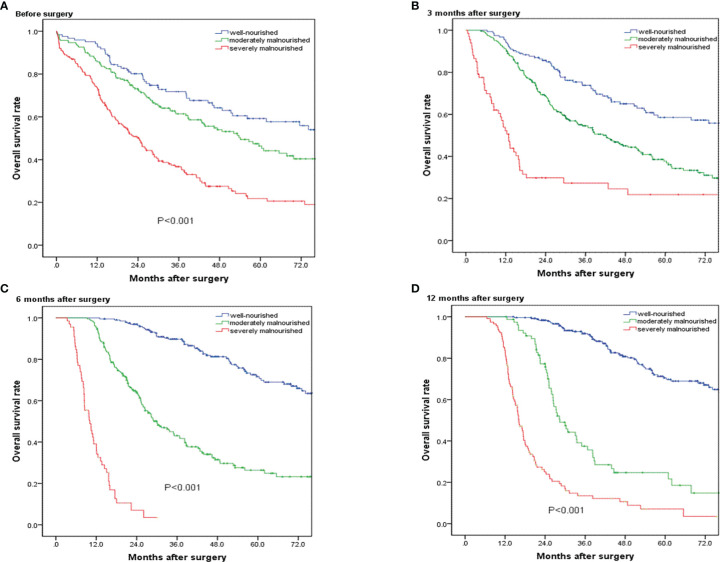
Overall survival curves for patients in the well-nourished group, moderately malnourished group, and severely malnourished group before and at 3, 6, and 12 months after pancreatoduodenectomy. **(A**–**D)** Significant overall survival difference were observed among the three groups (p < 0.001).

**Figure 3A** presents the Kaplan–Meier survival curves according to nutritional status before operation. The median overall survival time was 87.4 months in well-nourished group, 54.1 months in moderately malnourished group, and 24.3 months in severely malnourished group. Regarding overall survival, there was a significant survival difference between the three groups (p < 0.001). **Figures 3B–D**) presents the Kaplan–Meier survival curves according to nutritional status at 3, 6, and 12 months after operation, showing significant survival difference between the three groups. The results of univariate and multivariate Cox proportional hazards regression model showed that the preoperative PG-SGA score >9 scores was an independent prognostic factor in patients who underwent PD for ampullary carcinoma [hazard ratio (HR) = 1.623; 95% CI, 1.217–2.165; p < 0.001]. The results of univariate and multivariate Cox proportional hazards regression models for prognostic factors for OS are shown in **Table 4**.

**Table 4:** Univariate and multivariate Cox proportional hazard regression analysis of prognostic factors for OS in patients who underwent pancreatoduodenectomy for ampullary carcinoma after PSM.

Factors	No. Patients	Univariate analysis		Multivariate analysis		
		HR (95%CI)	P	HR (95%CI)		P
Age (>65 years vs. ≤65 years)	80/320	1.474 (1.098–1.978)	0.01			
Sex (male vs. female)	235/165	0.928 (0.720–1.196)	0.564			
Comorbidities (+ vs. −)	96/304	1.010 (0.754–1.353)	0.948			
BMI (>25 kg/m2 vs. ≤25 kg/m2)	47/353	1.013 (0.683–1.501)	0.950			
ASA class (III vs. I or II)	55/345	1.387 (0.981–1.961)	0.064			
T stage (T3/T4 vs. T1/T2)	206/194	1.831 (1.418–2.365)	<0.001				
Lymph node metastasis (+ vs. −)	148/252	2.054 (1.595–2.647)	<0.001			
Vascular invasion (+ vs. −)	31/369	1.903 (1.238–2.926)	0.003			
Perineural invasion (+ vs. −)	49/351	1.857 (1.299–2.655)	0.001	1.958 (1.307–2.934)		0.001
Tumor grade (poorly vs. moderately or well)	78/322	3.115 (2.316–4.190)	<0.001	2.280 (1.568–3.314)		<0.001
Resection margins (+ vs. −)	22/378	3.163 (1.985–5.042)	<0.001	2.768 (1.504–5.093)		0.001
TNM stage (III vs. I or II)	163/237	2.290 (1.776–2.954)	<0.001	1.575 (1.123–2.209)		0.008
Adjuvant therapy (+ vs. −)	146/254	0.588 (0.449–0.769)	<0.001	0.712 (0.520–0.976)		0.035
PG-SGA score						
Before operation (>9 vs. <9 scores)	200/200	1.840 (1.429–2.369)	<0.001	1.508 (1.103–2.061)		0.01
At 3 months after operation (>9 vs. <9 scores)	67/420	2.996 (2.146–4.183)	<0.001			
At 6 months after operation (>9 vs. <9 scores)	68/406	13.754 (9.449–19.995)	<0.001	4.148 (2.523–6.820)		<0.001
At 12 months after operation (>9 vs. <9 scores)	116/323	8.680 (6.314–11.931)	<0.001	5.272 (3.630–7.656)		<0.001

OS, overall survival; PSM, propensity score matching; BMI, body mass index; ASA, American Society of Anesthesiologists; PG-SGA, Patient-Generated Subjective Global Assessment.

### Univariate and Multivariate Cox Analysis of Prognostic Factors for Overall Survival

After PSM analysis, the results of univariate and multivariate Cox proportional hazards regression model showed severe malnutrition (PG-SGA score >9 scores) before operation (HR = 1.508; 95% CI, 1.103–2.061; p = 0.01) and at 6 months (HR = 4.148; 95% CI, 2.523–6.820; p < 0.001) and 12 months (HR = 5.272; 95% CI, 3.630–7.656; p < 0.001) after operation were independent prognostic factors in patients who underwent PD for ampullary carcinoma. Additionally, the positive perineural invasion (p = 0.001), poorly tumor grade (p < 0.001), positive resection margins (p = 0.001), TNM stage III (p = 0.008), and adjuvant therapy (p = 0.035) also were the independent prognostic factors of OS. The results of univariate and multivariate Cox proportional hazards regression model for prognostic factors for OS are shown in **Table 4**.

## Discussion

In recent years, the prognostic factors of postoperative outcome after PD have been extensively studied. The postoperative outcomes of patients with malignant tumors after curative surgery were associated with the tumor characteristics, surgical factors, and host-related factors (2–4). Among them, preoperative malnutrition was also an important risk factor of postoperative outcomes for malignant tumor after radical surgery (7, 13). Preoperative malnutrition in patients who underwent PD was often caused by the decrease in oral food intake, absorption disorder, and gastrointestinal obstruction. Patients who underwent PD frequently experience impaired digestive function that can affect long-term nutritional status. The consequences of preoperative and postoperative malnutrition on postoperative outcomes include increased incidence of complications, prolonged hospital day, and poor survivals. However, the studies examining whether postoperative malnutrition affects the long-term survival of patients who underwent PD for ampullary carcinoma were lacking.

In our study, the incidence of postoperative complications was more higher in severely malnourished group than in well-nourished or moderately malnourished group. The multivariate Cox proportional hazards regression analysis showed that severe malnutrition (PG-SGA score > 9 scores) before operation and at 6 and 12 months after operation were independent prognostic factors in patients who underwent PD for ampullary carcinoma.

As previously reported (11, 28), malnutrition was common in hospitalized patients with malignant tumor. Song et al. (11) analyzed the nutritional status of 23,904 patients with 16 common malignant tumors; 13,826 participants (57.88%) were moderately or severely malnourished. Similarly, most of the patients with ampullary carcinoma had varying degrees of malnutrition before and after operation in our study. Before operation, 122 (23.9%) patients were classified into well-nourished group, 189 (37.0%) into moderately malnourished group, and 200 (39.1%) into severely malnourished group. Additionally, many patients still suffer from malnutrition after operation; the median PG-SGA scores were 5, 4, and 3 scores at 3, 6, and 12 months after PD, respectively.

The nutritional status of hospitalized patients could be assessed by various methods, included objective anthropometric measurements, and subjective measurements based on the clinical history and physical examination (29–31). Among the aforementioned, the PG-SGA was the widely recommended method for the assessment of nutritional status of the tumor patients (17, 19, 32). The scored PG-SGA consists of a quantitative score and a qualitative global assessment, which was more sensitive to a subtle changes in nutritional status than the SGA. The higher the PG-SGA score is, the greater the risk of malnutrition is. Many previous studies have demonstrated that the scored PG-SGA was an effective and reliable tool for nutrition evaluation. Additionally, the NRS-2002 was also the preferred subjective screening tool for nutritional risk. According to the previous study, the PG-SGA scale is simple and accurate and may be the most suitable method for evaluating the nutritional status of malignant tumor patients and was more sensitive to the nutritional status in the general population of cancer patients compared with the NRS-2002.

Malnourished patients with malignant tumor often coexists with systemic inflammatory response and immunosuppression (14, 33). As previously reported (34, 35), the malignant tumors incite a systemic inflammatory response, which often triggers the release of inflammatory cytokines and chemokines, such as tumor necrosis factor and IL-1 and IL-6. Meanwhile, the systemic inflammatory response induced profound catabolic effects on host metabolism, for instance, which caused the depletion of skeletal muscle and visceral function proteins. In addition, neutrophilia and lymphocytopenia were commonly involved in cancer-related inflammation (36–38). Patients with immunosuppression and inflammatory response have poor tolerance to operation, recover slowly after surgery, and more susceptible to postoperative complications. Previous reports have found a relationship between preoperative malnutrition and poor short-term outcome and long-term survival, which were consistent with our results. In our cohort, we found that the incidence of postoperative complications in malnourished group was significant higher than that in the well-nourished group, and the preoperative malnutrition was the independent risk factor of overall survival.

To date, the relationship between postoperative long-term nutritional status and survival remains unknown. Previous studies reported that malnutrition after gastrectomy significantly and adversely affects overall survival; nutritional interventions to lessen the impact of postoperative malnutrition can offer hope for prolonged survival. However, they could not completely adjust for the negative survival impact of patient backgrounds in the malnutrition group and excluded patients with gastric cancer recurrence. In our study, to control for the effects of confounding factors, propensity score matched analysis was performed to balance the clinicopathological variables between preoperative group A (well-nourished and moderately malnourished group) and group B (severely malnourished group). After PSM analysis, we revealed relationships between poor survival and malnutrition before and at 6 and 12 months after PD. The PG-SGA score >9 scores before operation and at 6 and 12 months after operation were independent prognostic factors in patients who underwent PD for ampullary carcinoma. However, the PG-SGA score >9 scores at 3 months after operation was not the independent prognostic factor in our study.

In addition, as previously reported, the loss of skeletal muscle (SM) and visceral functional proteins was considered as the important indication of nutritional status (31, 39). The SM and visceral functional protein depletion leads to changes in intra- and extracellular electrolyte regulations and reduction in the metabolically active tissue and often coexists with immunosuppression, which were the important mechanisms for increased postoperative complications and poor survival after PD. de Oliveira Pereira et al. (39) quantitatively calculated the body composition using computed tomography and assessed the nutritional status of hospitalized patients, and they found that the PG-SGA was closely correlated with the skeletal muscle index. Meanwhile, compared with the weight loss and BMI, the scored PG-SGA was a more sensitive and precise method to assess nutritional status of patients with malignant tumor. Laky et al. (31) showed that BMI and percentage of weight loss were not able to effectively detect malnutrition of patients with malignant tumor because they could be masked by edema and dehydration. Similarly, Wigmore et al. (40) found that the median BMI of 20 pancreatic cancer patients at diagnosis was 20.7 (range, 19.5–23.6) kg/m^2^ despite a median weight loss of 14.2 (range, 10–20%) in the previous 6 months.

Besides, malnourished cancer patients were often accompanied by vitamin and microelement deficiency due to decreased oral food intake and impaired absorption. The deficiency of vitamin and microelement was closely related to symptoms such as nausea and vomiting and indigestion, which maybe the reason that the incidence of postoperative DGE after PD was more higher in malnourished group than in well-nourished group in our study. Regrettably, the incomplete data of vitamin and microelement test results makes it impossible to analyze its effect on postoperative DGE in this study.

These findings implied that nutritional status was associated with the postoperative outcomes of patients after PD. Therefore, preoperative and postoperative adequate nutritional support including oral or intravenous nutritional supplementation were considered important treatment means to improve the postoperative outcome of patients underwent PD. A retrospective multicenter study suggested that nutritional support before surgery might lead to better surgical outcome of resectable pancreatic cancer patients (41). Similarly, Adiamah and his colleagues found that preoperative immune modulating nutrition (IMN) had the significant impact on infectious complications and a tendency to shorten length of stay; preoperative IMN should be encouraged as a routine practice in patients undergoing surgery for gastrointestinal cancer (21).

This study has some limitations. First, it was retrospective and single-centered; thus, susceptible to the inherent problems of such design features, subject selection bias was unavoidable. In the future, prospective studies with larger sample sizes, and external validation of our findings in other populations, are essential.

## Conclusion

Our study suggested that patients with ampullary carcinoma had varying degrees of malnutrition before and after operation. Malnutrition before and at 6 and 12 months after PD significantly affects the long-term survival of patients. Additionally, the preoperative malnutrition was related to postoperative complications after PD. Nutritional interventions to lessen the impact of postoperative malnutrition might offer hope for prolonged survival.

## Data Availability Statement

The original contributions presented in the study are included in the article/supplementary materials. Further inquiries can be directed to the corresponding authors.

## Ethics Statement

All patients provided written informed consent to participate in the study, and the study was approved by the Ethics Committee of the local hospital (no. TJ-IRB20190418).

## Author Contributions

Conception and design: JJ, RQ, and GX. Administrative support: RQ and MW. Provision of study materials or patients: JJ and RQ. Collection and assembly of data: JJ, GX, and XW. Data analysis and interpretation: JJ. Manuscript writing: all authors. All authors contributed to the article and approved the submitted version.

## Funding

This study was supported by grants from the National Natural Science Foundation of China (Nos. 81772950 and 81874205). This study was also supported by a grant from the National Natural Science Foundation of China Youth Fund (No. 81902499).

## Conflict of Interest

The authors declare that the research was conducted in the absence of any commercial or financial relationships that could be construed as a potential conflict of interest.

## Publisher’s Note

All claims expressed in this article are solely those of the authors and do not necessarily represent those of their affiliated organizations, or those of the publisher, the editors and the reviewers. Any product that may be evaluated in this article, or claim that may be made by its manufacturer, is not guaranteed or endorsed by the publisher.
